# Understanding Necrosol pedogenetical processes in post-Roman burials developed on dunes sands

**DOI:** 10.1038/s41598-022-14750-5

**Published:** 2022-06-23

**Authors:** Zaira García-López, Antonio Martínez Cortizas, Noemi Álvarez-Fernández, Olalla López-Costas

**Affiliations:** 1 CRETUS, EcoPast (GI-1553), Universidade de Santiago de Compostela, 15782 Santiago de Compostela, Spain; 2 CRETUS, EcoPast (GI-1553), Area of Archaeology, Department of History, Universidade de Santiago de Compostela, 15782 Santiago de Compostela, Spain; 3 Archaeological Research Laboratory, Stockholm University, Wallenberglaboratoriet, 10691 Stockholm, Sweden

**Keywords:** Geochemistry, Fluorescence spectroscopy, Infrared spectroscopy, Biogeochemistry

## Abstract

In Archaeology much emphasis is dedicated to bone preservation, but less attention is paid to the burial soil (i.e., Necrosol), despite its crucial role in governing the geochemical environment. The interaction between human remains and sediments starts after inhumation, leading to bidirectional physico-chemical changes. To approach these complex, bidirectional processes, we sampled at high resolution (n = 46) two post-Roman wooden coffin burials (one single and another double), and the coeval paleosol (n = 20; nearby pedo-sedimentary sequence). The samples were analysed for physical (grain size, colour) and chemical (pH; LOI; elemental composition: FTIR-ATR, XRF, C, N) properties. Principal component analysis enabled to identify five main pedogenetical processes: decalcification, melanization, acidification, neoformation of secondary minerals (i.e., clays) and enrichment in phosphorus. Melanization, acidification and phosphorous enrichment seem to be convergent processes in Necrosols—irrespective of the parent material. Decalcification may be restricted to carbonate containing soil/sediments. Despite not mentioned in previous research, clay formation might also be an overall process. Compared to the local, coeval paleosol, pedogenesis in the studied burial soils was low (double burial) to moderate (single burial). Our results also emphasize the need to study the finer soil fractions, as they provide clues both on soil formation and bone diagenesis.

## Introduction

Necrosol is a valuable archive of pre- and post-mortem information. This term coined by Graf^1^ in 1986 refers to cemetery and burial soils. In the second half of the twentieth century, studies of Necrosol began to be developed, but it was not until 2004 that it was first described as: *Soils formed by special human activity in cemeteries and burial ground with specific soil horizons, specific physical, chemical and biological properties* (p. 110)^2^. Necrosol formation results from the interaction of the soil with human remains and other materials associated with the burial, the presence of a human body and skeletal remains that decompose/alter in-situ being key for this soil to be named. Changes taking place in the sediment happen both at short and long-term scales, concurrently with the body’s taphonomic processes^3^, turning it, most of the times, into a soil of rapid formation. After burial, flesh decomposition produces chemical compounds and physicochemical reactions that modify the surrounding soil/sediment. Once the body is skeletonized the alteration persists due to the direct contact between skeleton and soil/sediment, involving the diagenesis of bones and the pedogenesis of the Necrosol. While bone diagenesis is a well-stablished topic in the research agenda, especially in archaeological sciences^4–8^, the Necrosol has been hardly addressed.

Since bones incorporate elements by absorption and release them through chemical alteration, the pedogenetical/geochemical environment of the local soil/sediment influences bone preservation^7^. The importance of identifying pre-mortem acquisition from post-mortem change promoted the first studies on soils from archaeological burials. In 1988, Pate and Hutton^9^ analysed the exchange of chemical elements between inhumated bone and its associated sediments. A year later, Pate et al.^10^ emphasized the importance of the geochemical properties of burial sites and proposed a protocol for soil sampling during excavation. They also recommended taking samples from excavation profiles as to compare the general soil chemistry of the site with localised conditions in areas more adjacent to skeletons. From the 1990s onwards, more studies about Necrosol physicochemical properties have been published, mainly focused on the chemical properties^11–14^ and organic content^15,16^. Studies on the inorganic chemical composition only deal with a few elements (see^17^). In Archaeology, the increase of phosphorous content in soil was traditionally researched as a signal of skeletal remains or to identify a burial site^18–23^.

Today, Necrosol research is focused on present-day cemeteries^24–27^ or war mass graves^22,28–30^. Metal contamination is of major concern due to the ecological impact of large inhumation cemeteries^25,28,31^. Another growing research line concerns forensic cases^32^. Recent experiments simulate burials to analyse soil properties and animal tissue decomposition under controlled conditions^33,34^. Current studies are getting more complex and include a larger number of chemical elements ^30,35,36^, combining them with the study of soil micromorphology^15,37,38^ and complement bone diagenesis research with soil analysis^39,40^.

Although Necrosol has been described more than thirty years ago, to the best of our knowledge, no specific studies deal with its pedogenetic processes. As Lambert et al.^41^ stated, there are numerous investigations in soil chemical composition but not too many in soils associated to inhumated bones. For archaeological contexts to date, we have neither a complete description of Necrosol characteristic properties nor a standard approach for its characterization. Thus, present research applies different methodologies, analysing only specific characteristics or discussing single properties, most of the time as complement to the study of human remains. But understanding Necrosol formation has an important archaeological meaning. As Pickering et al.^16^ point out, Necrosol is a valuable archive of pre- and post-mortem information. Given that bone diagenesis acts differently depending on the geochemical environment (see a summary in^7^), it is necessary to reach a good comprehension of the Necrosol environment to be able to understand the archaeological context of inhumations and cremations.

**Figure 1:**
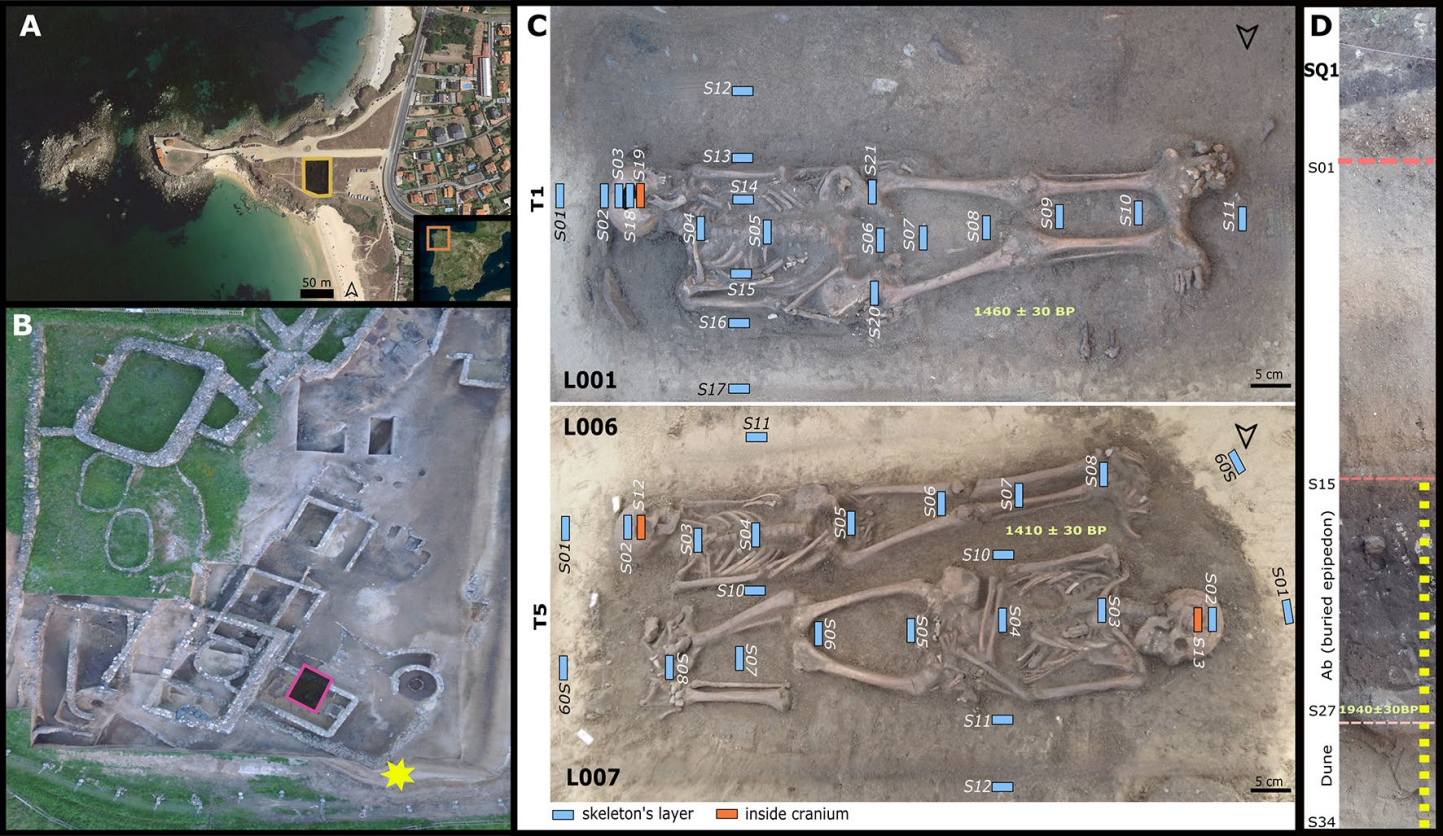
(**A**) and (**B**) Aerial view of A Lanzada with the burial area marked by a square and the location of the pedo-sedimentary sequence by a star in (**B**) (**A** modified from^42^, https://bit.ly/3FwpZrE; **B** modified from^43^, https://bit.ly/3BBqxKy). (**C**) Sampling design of T1 and T5 burials (image by Aerorec S.L. & Deputación de Pontevedra modified from^44^). (**D**) The pedo-sedimentary sequence and soil sampling (yellow dots; image by Aerorec S.L. & Deputación de Pontevedra modified from^44^). Radiocarbon datings are included in (**C**) and (**B**).

It may be important to approach the signal of Necrosol formation as part of the heritage they left, in particular in areas where the predominant acidic soils do not allow the preservation of skeletons. To approach these aspects, the present study aims to describe the characteristic composition and pedogenesis of Necrosol through a multi-sampling study of two post-Roman burials from NW Spain. To this end, we collected and analysed 46 samples from two burials: one containing and individual and another with two individuals; both originally in wooden coffins. Sampling was done in two transects in every individual, transvers and longitudinal; samples outsides the burials were also collected. The three skeletons were found in supine position, burials were West–East oriented (Fig. 1). We also sampled the lower soil cycle (n = 20) of a reference pedo-sedimentary sequence located 10 m from the burials. The sequence was composed of 3 main stratigraphical layers, each corresponding to a dune deposit and, thus, dominated by sands with biogenic carbonates. The two upper layers showed weak soil pedogenetical transformation and the bottom layer showed a moderate pedogenesis. The latter corresponded to the soil surface coeval of the burials and was composed of an upper buried epipedon (Ab horizon) and a lower layer of non-altered sands (C horizon)—in which the burials were made. The sampling design is described in detail in the “Material and methods” section. We also discuss to which extent the study of Necrosol can help to get insights into aspects of past societies and serve as a complementary source of information to the study of the human skeletal remains.

## Results

### Physicochemical properties (colour, grain size, pH)

Samples collected outside the burials were lighter and had higher hue (dune sands, L*: 71.7 ± 3.2; C*: 12.7 ± 1.8; h: 80.9 ± 1.9) than samples collected inside the burials (Necrosol, L*: 61.8 ± 4.4; C*: 14.3 ± 0.9; h: 79.1 ± 0.9) (SI_Fig. 1). However, both had a larger yellow than red component (yellow, b*: 13.7 ± 1.2; red, a*: 2.5 ± 0.5). Results from grain size analysis indicates a predominance of sand fractions, being ~ 85% in Necrosol and more than 95% in samples outside the burial. In addition, Necrosol has higher content of gravel (2.76 ± 1.7%), fine sands (22.22 ± 1.5%) and silt + clay (9.79 ± 2.81%) compared to samples outside the burials (gravel:1.46 ± 2.14%; FS: 19.62 ± 3.81%; SC: 2.32 ± 1.62%) (SI_Fig. 1). pH values reflect alkaline conditions, although samples from dune sands had larger values (9.3 ± 0.1 in water, 8.8 ± 0.2 in KCl) than those of the Necrosol (8.9 ± 0.2 in water, 8.3 ± 0.2 in KCl) (SI_Fig. 1).

Both soil horizons of the paleosol (Ab-C) had different characteristics. The colour of the buried epipedon (Ab) was dark brown; being a*, b* and chromaticity higher (a*: 3.3 ± 0.8; b*: 15.7 ± 1.7; C*: 16.0 ± 1.9) than in the dune sands (C horizon) (a*: 1.7 ± 0.4; b*: 11.2 ± 1.3; C*:11.4 ± 1.3). Whereas luminosity and hue were lower in the Ab (L: 63.5 ± 1.9 and h: 78.0 ± 1.4) compared to the C horizon (L: 75.8 ± 4.0 and h: 81.4 ± 1.12) (SI_Fig. 1). Grain size showed a predominance of sands in both horizons (more than 70%), although medium sands were more abundant in the C horizon, while coarse and fine sands were more abundant in the Ab. Gravel content was three times higher in Ab (19.6 ± 6.3%) than in C (6.0 ± 9.7). The difference is much larger for the silt + clay: the content in Ab (6.4 ± 3.3%) is about ten times higher than in C (0.6 ± 0.8%) (SI_Fig. 1). pH results indicate higher alkalinity in C (9.4 ± 0.1) than in Ab (8.9 ± 0.1), being pH very homogeneous within each soil horizon.

### Elemental composition (LOI, C, N and XRF)

The chemical composition is represented in SI_Fig. 1. Necrosol’s LOI (0.68 ± 0.15%) and N content (0.03 ± 0.01%) were higher than those of dune sands (LOI: 0.37 ± 0.07%; N: 0.014 ± 0.006%) (SI_Fig. 1), while C showed the opposite (Necrosol: 3.36 ± 0.23%; outside burials: 3.96 ± 0.51%). Regarding the other elements, some of them (i.e., S and Si) showed high concentrations in punctual samples, while other (i.e., Al, K, or Cr) presented more homogeneous concentrations among samples. Silicon, Ca, Rb, Sr, Zr and U concentrations were higher in samples outside the burials. In contrast, P, Cu, Zn and Br concentrations were more elevated in Necrosol samples.

In the paleosol, LOI values were higher in the Ab (2.68 ± 0.57%) than in the C horizon (1.09 ± 0.37%). Nitrogen showed a similar distribution (Ab: 0.06 ± 0.02%; C: 0.022 ± 0.015%) (SI_Fig. 1) but carbon was lower in the Ab (1.7 ± 0.11%) than in the C horizon (4.19 ± 0.66%). The Ab also presented higher concentrations for Fe, Ti, Ga, Rb, Y, Pb, Th and Br, while the C horizon had higher S, Ca and Sr content. Phosphorus content was higher and Mn content lower in the Necrosol than in the Ab of the paleosol.

### Spectroscopic analysis (FTIR-ATR)

**Figure 2:**
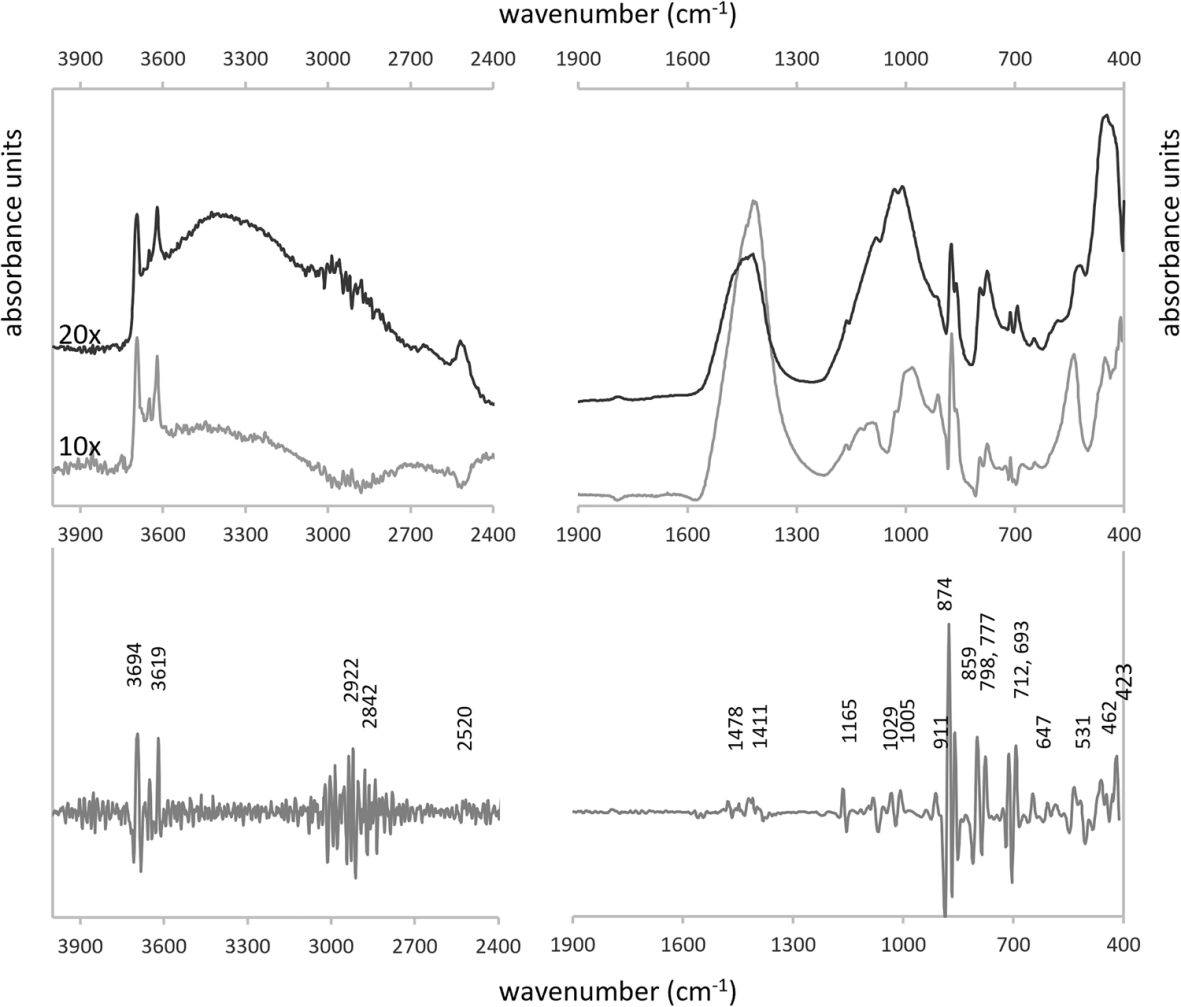
Top to bottom: average, standard deviation and average (reversed) second derivative spectra.

The average and standard deviation spectra, as well as the average spectrum of the second derivative are represented in Fig. 2. Six main absorbance areas can be observed: 3700–3400 cm^−1^, 2520–2510 cm^−1^, 1560–1300 cm^−1^, 1220–620 cm^−1^, and < 550 cm^−1^. The standard deviation spectra shows that variability between samples is largest in the 1560–1300 cm^−1^ region, very high in the regions 1050–850 cm^−1^ and 600–500 cm^−1^, and moderate to low in the regions 1200–1050 cm^−1^ and 3700–3600 cm^−1^. The second derivative spectrum (Fig. 2) enables to identified characteristic absorbances of soil components: quartz (1165, 1094, 1080, 798, 777, 693, 460 cm^−1^), K-feldspar (647, 535, 417 cm^−1^), carbonates (both calcite and aragonite, 2514, 1478–1411, 874, 859, and 712 cm^−1^), and clay minerals (i.e., kaolinite, 3694, 3668, 3647 and 3621, 1030, 1005, 911 cm^−1^); small amounts of mica (1005, 960, 527 cm^−1^) are also probable^45–47^. Very low absorbances around 3000–2800 and 1700–1600 cm^−1^ may correspond with low amounts of soil organic matter (SOM)^45,48,49^. While most of the vibrations in the region 1200–1050 cm^−1^ correspond to absorbances of silicate minerals, the standard deviation spectrum enables to identify a shoulder of moderate variability at 1200–1100 cm^−1^ that can be associated to vibrations of biogenic silica^50–54^.

**Figure 3:**
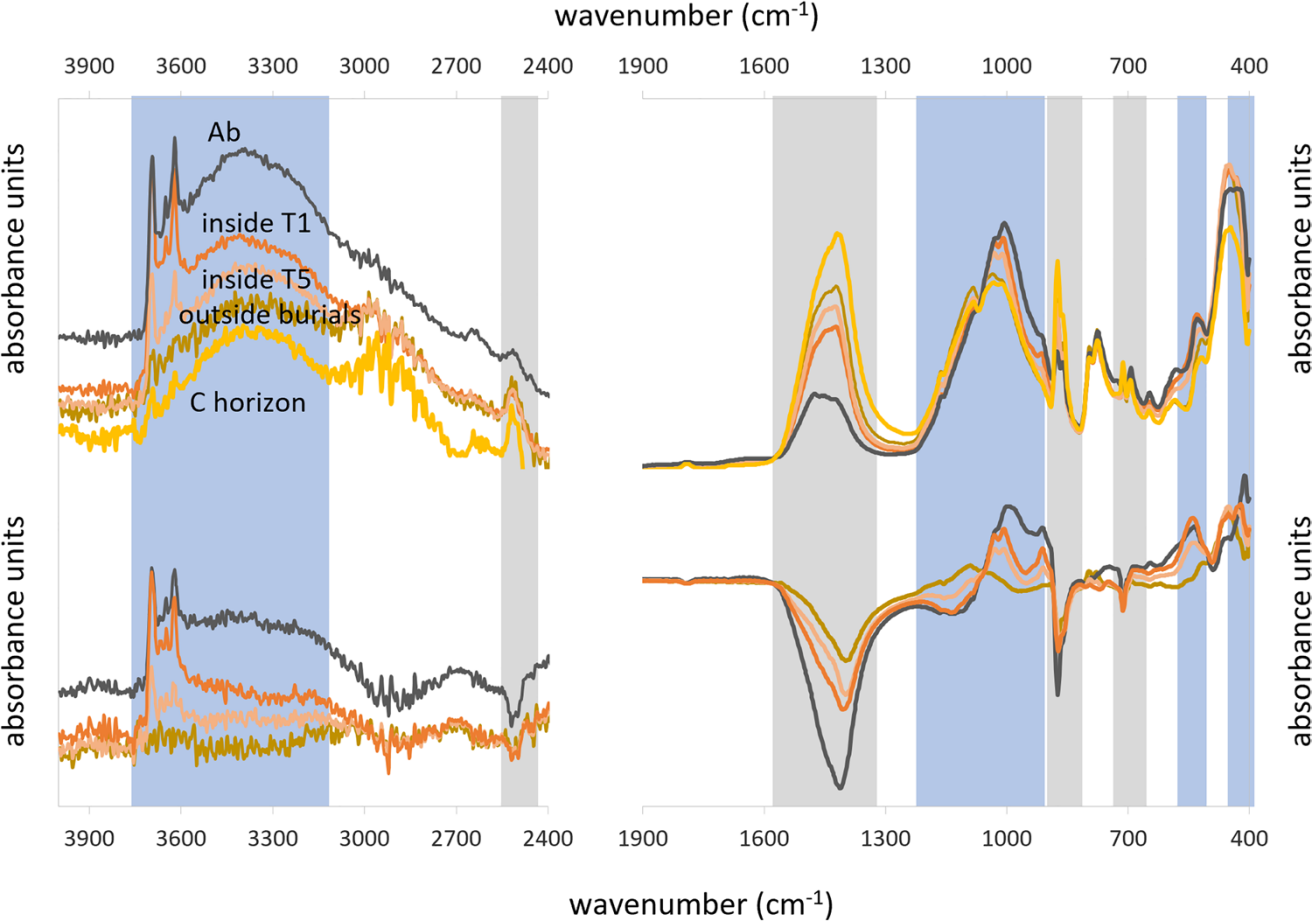
(**A**) Average spectra of the paleosol (black, Ab; yellow, C horizon), inside T1 (orange), inside T5 (pink), samples outside burials (dark brown). (**B**) Difference spectra compared to the dune sands, in blue the sections where the difference is positive and in grey where it is negative.

We computed difference spectra^55^ by subtracting the average spectrum of the dune sands samples (C horizon of the paleosol) to the average spectra of the Ab samples, the samples collected outside the burials and those from T1 and T5 Necrosol (Fig. 3). Negative differences are observed in the regions 2520–2510 (peaking at 2514 cm^−1^), 1560–1300 (peaking at 1411 cm^−1^), 900–850 (peaking at 874 and 859 cm^−1^) and 720–710 cm^−1^ (peaking at 712 cm^−1^). Positive differences are found in the regions 3700–3400, 1400–900, 600–500 and 480–420 cm^−1^. Negative differences correspond to carbonates’ vibrations, while positive differences correspond to quartz, clay and other silicates—and possibly also to SOM (OH vibrations around 3400 cm^−1^). Samples outside the burials do not show positive differences in the clay region (Fig. 3) and only low negative values for the carbonates’ region. The other samples showed an increasing trend in both negative values and positive values following the sequence: C horizon → outside burials → inside T5 → inside T1 → Ab horizon (Fig. 3).

### Principal components analysis

We performed a PCA using all analytical data (colour parameters, grain size, soil reaction, elemental composition, and selected absorbances of the IR data corresponding to soil components) obtained for the samples. The loadings of the 74 individual variables are in SI_Table 1. The first 5 components (73% of the total variance) contained at least a significant proportion of the variance of more than one variable and are the ones described here.

The first component, Cp1, accounted for 48.2% of the variance and showed large positive (> 0.7) loadings for kaolinite (3694, 3651, 3619, 1029, 1005, 911, 693, 647, 606, 585, 531, 423 cm-1) and OH (3424 and 3215 cm^−1^) vibrations, chromaticity and colour components (a* and b*), total SOM indicators (LOI, N), organically-bound elements (Br), and metal elements (Fe, Pb, Th, Ti) (SI_Table 1). Moderate (0.3–0.7) positive loadings were also shown by the silt + clay fraction, silicate IR absorbances (431 cm^−1^), SOM absorbances (aliphatic SOM: 2922, 2879, 2853, and 2842 cm^−1^), and some major (Al) and metallic (Rb, Y, Ga, Cu, and Zn) elements (SI_Table 1). Variables with large (< − 0.7) negative loadings include carbonates’ absorbances (1478, 1448, 1411, 874, 859, 712 cm^−1^), biogenic silica absorbances (1247, 1204, 1165, 1142, 1114 cm^−1^), hue (h) and luminosity (L*), soil reaction (pHw, pHk), total C, Ca, S and Sr, and medium and fine sands (SI_Table 1). Moderate (-0.43 to -0.65) negative loadings were also found for quartz absorbances (1081 and 1094 cm^−1^) and U.

Cp2 accounted for 9.9% of the total variance, coarse sand and Zr showed a large positive loading, while P had a large negative loading (SI_Table 1). Moderate positive loadings were also found for many elements (Y, Rb, Mn, Si, Fe, Nb, N, Br, Pb), carbonates (i.e., aragonite, 859 and 712 cm^−1^), L*, soil reaction (pHw), and aliphatic SOM (2922 cm^−1^). Moderate negative loadings were found for fine soil components (silt + clay), silicates (quartz and kaolinite, 532, 449, 431 and 423 cm^−1^), Ca and Cu (SI_Table 1).

Cp3 (9.3% of the total variance) is dominated by large to moderate positive loadings of quartz (1094, 1081, 798, 777, 462, 431 cm^−1^), aliphatic SOM (2922, 2879, 2853, 2842 cm^−1^), biogenic silica (1165, 1142, 1114 cm^−1^), K and Rb. Carbonates (i.e., calcite and aragonite, 1411, 874, 859, 712 cm^−1^), clays (i.e., kaolinite, 3694, 911, 647, 606, 585, 531 cm^−1^) and S, have moderate negative loadings (SI_Table 1).

Cp4 (3.5% of total variance) shows no large loadings. Moderate positive loadings were obtained for Al, Ti, K, Si, U and colour components (a*, b* and chromaticity) and moderate negative loadings for Cr, Zn and hue (SI_Table_1). Cp5 (2% of total variance) is dominated by the anti-covariation of Cr, Si and U against Zn and Fe (SI_Table 1).

**Figure 4:**
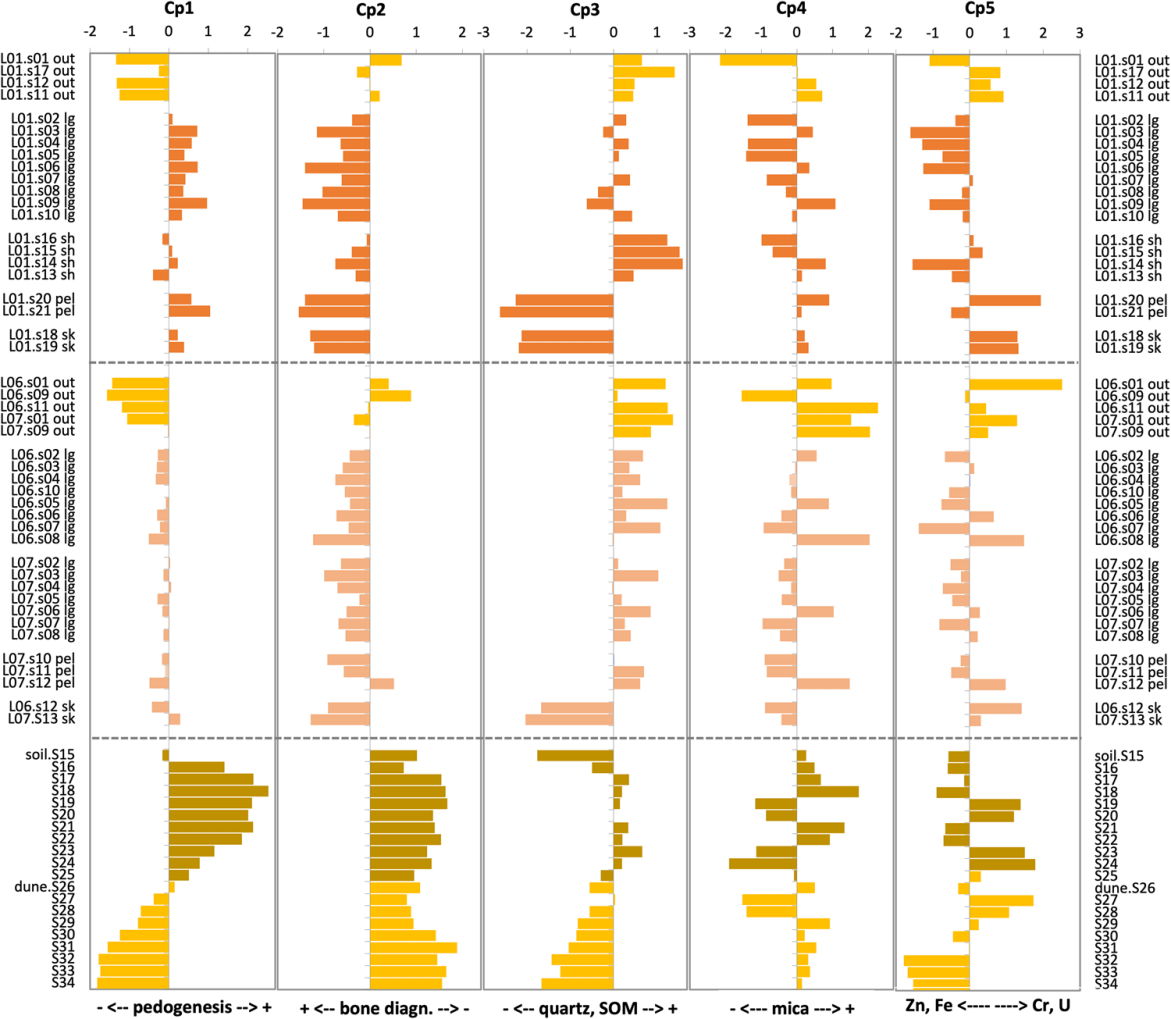
PCA scores of the analysed samples (burials, Ab-soil, and dune-C horizon), for the five main principal components. The burial samples have been grouped according to the place they were taken (*out* outside the burial), the transect (*lg* longitudinal) and the anatomical region (*sk* skull, *sh* shoulders, *pel* pelvis) with which they were related.

Figure 4 shows the components’ scores of the samples of the burials (outside and inside), and the paleosol. Positive Cp1 scores are found for the Ab and most of the samples from inside T1. The C horizon and samples collected outside the burials show negative loadings. Regarding Cp2, all samples from the paleosol have positive scores while almost all samples collected inside and outside the burials show negative scores, exception made of a few samples outside the burials (Fig. 4). Cp3 scores show an increase in value from the C horizon to the buried epipedon, to decrease again at the top of this horizon; samples from Necrosol show no clear trend, although those collected in the skulls have large negative scores while the rest have positive or slightly negative scores. No pattern is observed for Cp4 and Cp5 scores, both in the paleosol (Ab and C horizon) and burial samples (Fig. 4).

## Discussion

**Figure 5:**
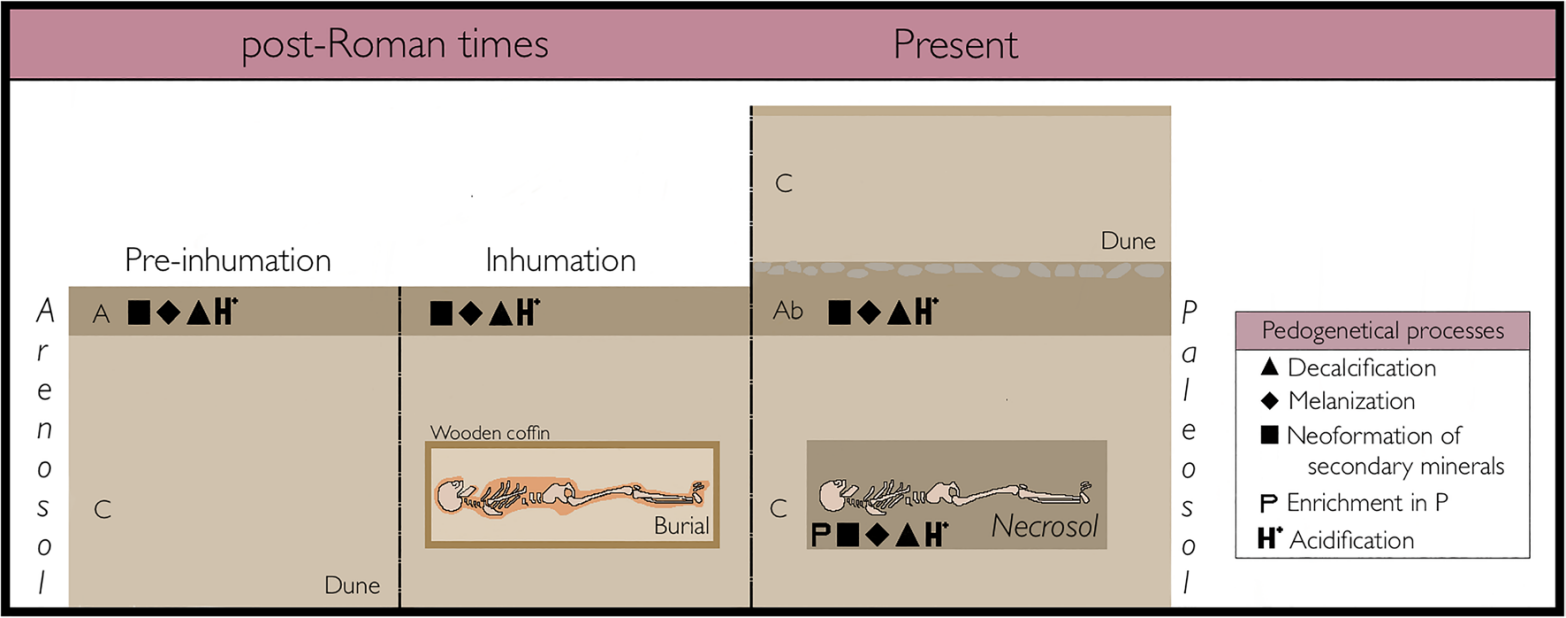
Diagram of the Necrosol pedogenesis compared with that of the buried epipedon of the Arenosol.

The extracted principal components represent the five major geochemical signals that characterize the the paleosol (Ab-C horizons; Arenosol), the Necrosol and the samples outside the burials (i.e., corresponding to the C horizon of the Arenosol) (see Fig. 5). The results indicate that the first three components are the ones that capture the main differences between the burials’ Necrosol and the contemporaneous soil, while the other two seem to reflect small-scale heterogeneity in the distribution of certain soil components (i.e., mica and metals).

### Degree of pedogenesis

The first principal component, Cp1, is the one that accounts for a larger number of soil properties. Properties with positive loadings are essentially related to the enrichment in secondary minerals (kaolinite and silt + clay fractions), SOM (total SOM, N, aliphatic SOM) (SI_Table 1) and changes in soil reaction (i.e., pH). Properties with negative loadings are related to the abundance of biogenic carbonates (calcite, aragonite, C, Ca and Sr) and biogenic silica. Thus, the component is reflecting the main pedogenetic processes occurring in the palesol and the Necrosol (Fig. 5): decalcification (i.e., weathering of biogenic carbonates), melanization (SOM accumulation), and neoformation of secondary minerals (i.e., clay formation) (see^56^). The progression of pedogenesis is also accompanied by changes in other soil properties, as a decrease in soil luminosity and hue, and an increase in chromaticity (and both redness and yellowness), increased soil acidity (i.e., lower pH), and increased concentrations of metals and organically bound elements (Al, Fe, Ti, Rb, Cu, Ga, Y, Pb, Zn, Br) (SI_Table 1). Carbon, usually correlated to N content in soil due to both being basic constituents of the SOM, is here decoupled to SOM content because the inorganic C of the biogenic carbonates (e.g., from shells) dominates the C pool. In a similar way, S seems to be more dependent on the biogenic carbonates that on SOM.

The scores of Cp1 can be taken as a measure of the degree of pedogenesis: negative loadings indicating no or only slight pedogenetic evolution and positive loadings indicating more intense pedogenetic evolution. The dune sands (C-horizon of the paleosol and samples collected outside the burials) have a very similar composition and the largest negative scores. This indicates no or weak pedogenesis, a fact that means that both are parent material of the Ab horizon and the Necrosol respectively. The paleosol shows the typical evolution in soil profiles of increasing pedogenetic transformation from the parent material to the epipedon (i.e., the Ab horizon, in our case) (Fig. 5). The lower values at the top two samples of the paleosol are due to the transition to the overlying dune cycle that buried the epipedon (Fig. 4).

Cp1 scores show much lower variation in Necrosol, suggesting a lower degree of pedogenesis than that found for the Ab; but clear differences can be observed (Fig. 4). In T1 values are almost all positive, while in T5 values are almost all slightly negative. The intensity of pedogenesis in T1 is comparable to that of the lower half of the Ab, and in T5 it is comparable to that observed in the transition between the C horizon and the Ab (Fig. 4). In T1, samples collected in the shoulders transect show the weakest pedogenesis. No clear differences were found in T5. We should keep in mind that while pedogenetic transformations can be similar between the Arenosol and the Necrosol, the origin of both soils is not alike. In the first case, it was a surface layer, while the Necrosol is always a buried layer that contains a body in decomposition/alteration (Fig. 5). Our results point to pedogenetic convergence given the same parental material.

Comparing the main pedogenetic processes occurring in the Necrosol studied by us with others around the world, all share similar processes. Although ours is the first study of the Necrosol that used quantitative colour (with a colorimeter), melanization has been also found in previous studies. Fiedler et al.^22^ and Majgier and Rahmonov^12^ recorded darker colours in the Necrosol using Munsell scale (by human-eye). Other authors only indicate that the Necrosol is dark-coloured^16,36^. The dark colour of the Necrosol can be observed in the rectangular shape around the skeletons, and the finding of iron nails in the borders, pointing to the use of wooden coffins.

The alkalization of Necrosols has been suggested as a key factor for skeletal remains preservation^5^. However, all samples from the burials (T1 and T5) are less alkaline than those of the parent material (C-horizon), pointing to some degree of acidification -as it happens in the Ab of the paleosol. Although bone chemical weathering may contribute to soil alkalinization, the acidity generated by the enrichment in organic matter may counter-balance the effect through intense chemical weathering and leaching of the biogenic carbonate (i.e., decarbonation). This process seems to have been slightly more intense in the thoracic area of individual L006, which could be linked to his more confined decomposition (see “Material and methods” section). Here, the organic matter may have caused a greater acidification of the soil in contact with this skeleton. Acidification of the Necrosol has been observed by other researchers^11–13,30,57^ in soils originally alkaline, and was also linked to the increase in organic matter and the decrease in calcium content (decalcification). To approach the influence of soil pH on bone conservation, it must be also determined in the pre-burial soil or sampling surrounding areas, since the pH of the Necrosol is highly influenced by the soil-skeleton exchange over time.

Metal enrichment has been described in other investigations^28,35,36,57^, which found an increase in Al, Fe, Pb, Zn and Rb in the Necrosols. Keeley et al.^58^, Amuno & Amuno^28^ and Charzyński et al.^57^ found higher Cu concentrations inside burials. However, Charzyński et al.^57^ and Pankowská et al.^35^, in investigations of Necrosols with cremated remains from Nazi concentration camps, suggested that Cu concentrations could respond to other post-depositional processes.

In our study, the enrichment in metals and elements associated with organic matter (i.e., N and Br) is accompanied by an increase of fine (i.e., silt and clay) soil fractions. Most metal elements are enriched in the fine fractions of soils and sediments^59–61^. To date, not much attention has been paid to the differences in the content of silt and clay fractions inside and outside the burials. To our knowledge, only our previous investigation in Álvarez-Fernández, et al.^62^ analysed the silt and clay fraction apart from the fine earth. In this study, we encouraged the analysis of fine fractions, when studying sandy soil/sediments in particular, as they are the most reactive and have the largest potential to contain information about the interaction between buried bodies and burial environment. However, this is the first study in which an enrichment in silt and clay was observed inside the burials.

### Enrichment in phosphorous

The second principal component is dominated by the inverse distribution of coarse sands and Zr (probably reflecting the content in zircon) regarding P content. This component accounts for large differences between the Arenosol and the Necrosol (Fig. 4). The Necrosol is enriched in P and shows a predominance of smaller grain sizes than the Arenosol. Thus, phosphorous enrichment seems to be a specific process occurring in the Necrosol (Fig. 5), which is certainly related to the chemical weathering of the human remains (both soft tissues and bone). Differences are also observed between burials, since T1 shows a larger enrichment than T5. As it was the case for the intensity of pedogenesis, in T1 the lowest P enrichment is observed in the shoulders transect, while distribution is more homogeneous in T5. It is somewhat surprising that the burial containing two individuals, and thus more initial body mass for P release, has lower average P concentrations than the burial with only one individual. Two complementary aspects should account for this result: (1) T1 is a more confined environment and a larger proportion of the products of body decomposition may have accumulated within the burial area; (2) the individual buried in T1 was a senile woman (> 60 years), affected by age-related osteoporosis, which may have resulted in enhanced bone chemical weathering.

Phosphorus is an element traditionally used in Environmental Archaeology to detect the presence of human activity^63–66^ and to identify burials^19^. Although there are other elements that humans transfer to the soils, phosphorus is the least susceptible to change and leaching, and therefore lasts over time^67,68^. For burials, phosphorous and calcium are the major elements of the bone mineral component (i.e., hydroxyapatite) that are released to the surrounding soil due to bone alteration. The process underlying Cp2 is probably the result of this release as shown by the correlation between phosphorus and calcium and silt + clay. The correlation with calcium could be explained because when phosphorus is released into the soil it binds to calcium, iron or aluminium to form stable inorganic compounds^67^. However, we only see correlation with calcium and not with iron and aluminium, most likely because of the alkaline nature of the parent material^66^ and the wide availability of calcium. These compounds help to keep phosphorus in the soil, but it has also been documented that in sandy soils, such as the Necrosol studied here, leaching does occur. What is different about the Necrosol that allows phosphorus to persist? The most likely component responsible for that is the finer soil fraction (i.e., clay), that is the most reactive. Numerous studies describe the enrichment of phosphorus in Necrosols^12,14,22,28,35,36^ and it seems an important specific feature of Necrosol pedogenesis.

### Secondary enrichment in resistant minerals and SOM

Component Cp3 captures a pedogenetic signal related to the accumulation of resistant minerals and resistant SOM compounds that is more intense in the Necrosol than in the Arenosol. In the paleosol, a clear trend (Fig. 4) of increasing accumulation of resistant minerals and SOM is observed from the C horizon to the upper part of the Ab—exception made of the upper two samples, for reasons already commented in the degree of pedogenesis (Fig. 4). This trend is consistent with an increase in the degree of pedogenesis. But, while the Ab shows low positive values, Cp3 scores in the burials’ soils are larger both outside and inside the burial, except for the samples taken in and around the skulls (T1 and T5) and the pelvis transect (of T1). This secondary enrichment in aliphatic SOM, which is more resistant to biological degradation than other organic compounds (as proteins and polysaccharides), is probably related to the decomposition of the body soft tissues and of the wooden coffins. At this stage, we do not have a proper explanation for the secondary enrichment in resistant minerals in the burials. But we cannot rule out that it derives from the digging and burying operations in post-Roman time—mixing of sand layers, deposition of external materials in the dune sands while the burials were open, etc.

### Soil/sediment heterogeneity

Components Cp4 and Cp5 are related to the content in some minerals (probably micas) and metals (Zn, Fe, Cr, U). Although scores tend to be negative inside the burials, reflecting a somewhat lower content of mica, Cr and U, but higher content in Zn and Fe, distribution patterns are not clear and may reflect micro-scale heterogeneity in the composition of the soil/sediment and in the geochemical conditions driving the release, mobility and accumulation of the metal elements (see for example^7^).

## Conclusions

This investigation aimed to characterize Necrosol pedogenesis and composition in two burials from post-Roman times (AD sixth century). Our results provide information on the main five processes that took place: decalcification, acidification, melanization, neoformation of secondary minerals and enrichment in phosphorus. As a result of these processes the Necrosol acquires some characteristics that describe it as having darker colour, lower alkalinity and higher content of fine particles, organic matter and phosphorus. Translated into archaeological information, by comparing the chemical composition of the Necrosol and the surrounding soil we determined that these two inhumations from A Lanzada were dug in the dune and the same material was used to cover the wood coffins; therefore, there was no reconditioning of the burial site. The decomposition of wood coffins and the individuals’ bodies together with bone diagenesis triggered the formation of the Necrosol.

Although many studies indicate the increase in phosphorus content as a key aspect of necropolises’ soils, our study reveals that other pedogenetic processes must also be considered. Melaninization and acidification are very characteristic of the Necrosol from the studied burials but also in the buried epipedon (Ab), which points to a common soil process related to the increase in SOM. Higher phosphorous content and increase in metals seem to be related to the diagenesis of the skeletons—in consequence, we would expect the same processes to occur when soil is in contact with the bone. Each pedogenetic process separately can be observed in other soil types, but their combination together with accumulation of resistant minerals and resistant SOM compounds are, in our opinion, the key for a Necrosol in sandy parent materials to be described. Although our results are promising and provide a suitable explanation for other observations made in the literature, more studies are needed considering different chronological frames and parent materials to better understand Necrosol formation. Our findings suggest that the study of the Necrosol complements the characterization of human remains, being both relevant to understand the burial ritual.

## Material and methods

### Location and sampling

The site of “A Lanzada” (Noalla, Sanxenxo) is located in the province of Pontevedra, NW Spain (UTM 51023814X; 4697448.14Y) (Fig. 1). It has been the subject of numerous archaeological campaigns from the 1950s, the last one in 2016–2017^44^. Archaeological remains point to a wide chronology of occupation from the eighth century BC (Late Bronze Age) to AD tenth century (Middle Ages)^69–72^. One of the most remarkable archaeological features of A Lanzada site is its necropolis (Fig. 1), with two well-defined funerary areas dated from Roman and post-Roman times^72^. The last archaeological campaign (2016–2017) was focused on the East area of the site (Fig. 1) in which, adjacent to a monumental size house (a possible religious building), two burials were excavated (by OLC and AMC): T1, with one individual (L01), and T5, with two individuals (L06 and L07) (Fig. 1). L01 was a senile age female (L01, > 60 years-old), while L06 was a male adolescent (13–20 years-old) and L07 a mature male (40–60 years-old). The three skeletons were buried in dune sands and a clear brownish colour, rectangular shape pattern was observed embedding them (Fig. 1). This fact, together with the presence of iron-made nails and slight displacements at join articulations, have been archaeologically interpreted as they were buried in wooden coffins. As it can be seen in Fig. 1, the skeletons were in supine position with stretched legs. L001 is West–East oriented with arms stretched along the body. L006 was also West–East oriented with arms stretched and hands over the pelvic area. L007 was East–West oriented, and his arms were crossed over the abdomen. Open space decomposition is noticeable in L001 and L007 due to the rotation of the innominate bones and the head of the femurs. L006 skeleton is more confined against the lateral of the coffin and most of his joins did not rotate.

We collected 46 samples from within both burials, using a multisampling design organised in two transects—longitudinal and transverse—along every individual. Additional samples (5) were also taken from inside the crania and the innominate bone area (Fig. 1). To contextualize the evolution of the site, a pedo-sedimentary sequence (SQ1) (Fig. 1), located 10 m away from the burial area, was also collected. For this study we selected 20 soil samples (5 cm in thickness) corresponding to the soil (i.e., paleosol) contemporaneous of the burials. The selected samples comprise two horizons: (1) a buried epipedon (Ab) that represents the surface of the soil that was in use when the burials were excavated, (2) the dune sands or underlying parent material (C horizon), that represent the layer where inhumations were placed. In a previous work^7^, we have classified the soils in the archaeological site as Haplic Arenosol (calcaric)^73^.

To determine the chronology of the paleosol and the burials, 14C dating was carried out. One soil sample (silt + clay fraction) of the sequence (SQ1.S27) and two samples of left ribs collagen of the individuals L01 and L06 were analysed. The results are presented in Fig. 1 and SI_Table 2 and indicate that the individuals were buried by the end of the sixth century AD and the buried epipedon may have started its formation in the first century AD.

### Physicochemical, elemental, and spectroscopic analyses

All samples (fine earth, < 2 mm) were analysed for physical (grain size, colour) and chemical (pH, LOI, elemental composition: C and N, XRF, FTIR-ATR) properties. Grain size analysis was performed using a set of sieves to determine the percentage of coarse sand (2–0.5 mm), medium sand (0.5–0.2 mm), fine sand (0.2–0.05 mm) and silt + clay (< 0.05 mm). Soil/sediment reaction (pH) was measured in water (pHw) and KCl (pHKCl) suspensions (ratio 1:2.5) with a pHmeter^74^. Colour was measured using a CR-5 Konica Minolta colourimeter, using the CIELab colour space that provides five parameters: L* (luminosity), a* (green–red component), b* (blue–yellow component), C* (chromaticity), and h (hue). Loss on ignition (LOI) was obtained to estimate soil organic matter content, by heating the samples at 105 °C for 24 h and then ashing at 550 °C for 5 h in a muffle furnace. Carbon and nitrogen content was measured using a LECO-TruSpec CHNS analyser; while the concentrations of P, S, Si, Al, Fe, Ti, K, Ca, Ga, Rb, Sr, Y, Zr, Nb, Cr, Mn, Ni, Cu, Zn, Pb, Th, U and Br were determined by X-ray fluorescence. The XRF equipment used were calibrated with standard reference materials and both are hosted at the RIAIDT facility of the Universidade of Santiago de Compostela, Spain. Quantification limits were 0.01% for major elements (Si, Al, Fe, Ti, K and Ca), 100 μg g^−1^ for P, S and Mn, 1 μg g^−1^ for Ga, Rb, Sr, Y, Zr, Nb, Cu, Zn, Th, Ni, Cr, U and Br; for Pb quantification limit was 0.5 μg g^−1^.

Spectra were acquired in finely milled samples, in the mid-infrared (MIR) region (4000–400 cm^−1^) by attenuated total reflectance (ATR), using an Agilent Technologies Cary 630 spectrometer. Resolution was set to 4 cm^−1^ and each spectrum is the average of 200 scans. The equipment was thoroughly cleaned before each measurement and a background was collected before every sample. Average, standard deviation and second derivative spectra and peak identification (based on the second derivative spectrum) were obtained with {andurinha} R package^75^. Assignment of compounds related to vibrations and classes is based on literature (see references in “Secondary enrichment in resistant minerals and SOM” section), considering the limitations imposed on IR interpretation of complex samples^49,76–78^.

### Statistical methods

Principal components analysis (PCA) was performed on the set of properties analysed (74 variables in 66 samples), in correlation mode and using a non-rotated solution. One factor ANOVA was run on the components scores, grouping according to the different groups that represent soils/horizons (inside and outside the burials, buried epipedon and dune sands). Since grain size data and elemental concentrations are a case of close data^79^, we applied a centred log-ratio (clr) transformation prior to statistical analyses^80^. Statistical analyses were made using software R^81^ (package {andurinha}^75^) and SPSS Statistics 23.

## Supplementary Information

Supplementary Information.

## Data Availability

All data generated or analysed during this study are included in this published article in the supplementary files.
