# Supplementary material for: Understanding Necrosol pedogenetical processes in post-Roman burials developed on dunes sands
Source: Sci Rep. 2022 Jun 23;12:10619. doi: 10.1038/s41598-022-14750-5 (PMC9226110; doi:10.1038/s41598-022-14750-5)
Supplement: Supplementary file 1 — Supplementary Information. [file 41598_2022_14750_MOESM1_ESM.pdf]

Understanding Necrosol pedogenetical processes in post-Roman burials developed on dunes sands

Zaira García-López, Antonio Martínez Cortizas, Noemi Álvarez-Fernández, Olalla López-Costas

Supporting Information

SI_Table 1. Loadings of the soil properties determined in the samples collected in the paleosol and burials T1 and T5 of A Lanzada archaeological site. Each component and their elements' loadings are identified with a colour (Cp1: yellow; Cp2: purple; Cp3: pink; Cp4: light blue and Cp5: green).

Soil component	variable	Cp1	Cp2	Cp3	Cp4	Cp5	Cp6	Cp7	Cp8	Cp9	Cp10
Kaolinite	423	0.75	-0.43	0.27	0.12	-0.09	-0.13	0.08	-0.08	-0.10	0.17
Kaolinite	531	0.73	-0.39	-0.47	0.19	-0.02	0.09	0.04	0.06	0.00	0.07
Kaolinite	585	0.91	0.04	-0.33	-0.09	0.10	0.04	0.02	-0.03	-0.11	0.06
Kaolinite	606	0.89	-0.03	-0.39	-0.01	0.08	0.08	0.00	-0.05	-0.11	0.05
Kaolinite	647	0.89	0.13	-0.32	0.04	0.14	0.12	-0.01	-0.12	-0.05	0.02
Kaolinite	693	0.81	-0.21	-0.04	0.14	0.23	-0.04	0.05	-0.19	0.11	-0.09
Kaolinite	911	0.91	0.05	-0.32	0.12	0.15	0.03	0.02	-0.03	0.08	-0.08
Kaolinite	1005	0.89	0.02	-0.17	-0.18	0.18	-0.24	0.11	0.02	-0.01	-0.02
Kaolinite	1029	0.84	-0.22	-0.12	-0.19	0.15	-0.33	0.14	0.07	-0.01	-0.04
OH	3215	0.89	0.14	0.24	0.05	0.05	0.19	-0.02	0.02	0.00	-0.04
OH	3424	0.90	0.13	0.21	-0.03	0.04	0.17	-0.02	0.04	-0.01	-0.02
Kaolinite	3619	0.91	-0.13	-0.24	0.04	0.07	0.05	0.08	0.15	0.12	-0.11
Kaolinite	3651	0.91	-0.08	-0.15	0.01	0.01	0.02	-0.08	0.20	0.05	-0.15
Kaolinite	3694	0.86	-0.18	-0.36	0.12	0.07	0.05	0.07	0.14	0.10	-0.04
Bromine	Br	0.78	0.41	-0.05	-0.24	-0.02	-0.20	-0.02	0.03	0.02	0.00
Chromaticity	chr	0.84	-0.18	0.09	0.38	-0.05	-0.12	-0.15	0.04	0.11	0.03
Iron	Fe	0.60	0.55	-0.01	-0.23	-0.38	0.05	0.13	0.16	0.11	-0.10
Total SOM	LOI	0.73	0.61	-0.11	-0.08	0.00	-0.08	0.04	-0.13	-0.11	0.02
Nitrogen	N	0.71	0.43	-0.07	-0.08	-0.04	-0.08	-0.13	-0.05	-0.07	-0.10
Lead	Pb	0.65	0.36	0.24	0.08	0.09	-0.18	-0.19	0.08	0.20	0.07
Redness	red	0.82	-0.17	0.07	0.40	-0.17	-0.08	-0.16	-0.11	0.07	-0.01
Thorium	Th	0.73	0.28	0.01	-0.18	-0.06	-0.14	-0.06	0.19	0.20	-0.05
Titanium	Ti	0.64	0.21	0.19	0.52	-0.03	-0.03	0.17	-0.16	-0.19	-0.19
Yellowness	yell	0.84	-0.19	0.09	0.38	-0.04	-0.12	-0.15	0.05	0.11	0.04
Carbonate	712	-0.66	0.40	-0.54	0.19	-0.03	0.05	-0.04	-0.18	-0.03	-0.02
Carbonate	859	-0.72	0.38	-0.50	-0.01	0.07	-0.11	0.07	-0.13	0.05	0.00
Carbonate	874	-0.89	-0.09	-0.41	-0.03	-0.10	-0.04	-0.04	0.02	-0.02	0.06

Biogenic Si	1114	-0.78	0.00	0.59	-0.05	0.03	-0.11	0.01	0.03	0.03	0.02
Biogenic Si	1142	-0.83	0.14	0.52	0.00	-0.03	-0.01	-0.05	-0.03	-0.01	0.03
Biogenic Si	1165	-0.85	0.11	0.48	0.04	-0.05	0.00	-0.05	-0.02	0.02	0.00
Biogenic Si	1204	-0.94	0.07	0.24	0.11	-0.13	0.04	-0.06	0.03	0.04	-0.07
Biogenic Si	1247	-0.87	0.21	-0.23	0.24	-0.17	0.12	-0.06	-0.01	0.03	-0.13
Carbonate	1411	-0.93	0.00	-0.33	-0.03	-0.11	-0.03	-0.04	0.06	0.01	0.02
Carbonate	1448	-0.93	-0.10	-0.27	-0.08	-0.11	-0.06	0.00	0.04	-0.03	0.10
Carbonate	1478	-0.94	-0.09	-0.22	-0.07	-0.09	-0.06	0.02	0.01	-0.03	0.13
Carbon	C	-0.95	0.00	-0.09	0.22	-0.08	-0.12	0.05	-0.01	-0.05	0.05
Calcium	Ca	-0.90	-0.33	-0.09	0.16	-0.08	-0.12	0.07	0.03	-0.01	0.07
Fine sand	FS	-0.63	0.07	0.16	0.19	0.10	0.47	0.39	0.04	0.09	-0.04
Hue	hue	-0.74	0.24	-0.04	-0.36	0.22	0.09	0.13	0.26	0.00	0.01
Luminosity	lum	-0.71	0.57	-0.01	0.03	0.12	0.01	0.05	0.18	0.06	0.19
Medium sand	MS	-0.93	-0.13	0.06	0.09	-0.02	0.18	0.01	0.10	0.14	-0.14
Soil reaction	pHk	-0.91	0.16	0.15	0.21	0.03	0.16	-0.03	0.02	0.10	-0.04
Soil reaction	pHw	-0.75	0.32	0.26	0.18	0.11	0.22	0.06	-0.08	0.07	-0.03
Sulphur	S	-0.79	-0.25	-0.36	0.19	0.06	0.05	0.12	0.17	0.03	-0.03
Strontium	Sr	-0.93	-0.08	-0.08	0.15	-0.11	-0.16	0.09	-0.04	-0.06	0.11
Coarse sand	CS	-0.25	0.94	-0.07	0.01	-0.02	-0.06	-0.02	-0.14	-0.07	-0.05
Gallium	Ga	0.39	0.51	-0.06	0.18	-0.27	0.23	-0.13	0.12	0.10	0.25
Manganese	Mn	0.05	0.63	-0.03	-0.02	-0.28	0.12	0.37	0.16	-0.13	-0.09
Niobium	Nb	0.22	0.43	-0.10	0.26	0.15	-0.16	0.16	0.37	0.27	0.23
Rubidium	Rb	0.54	0.65	0.31	0.17	0.09	-0.04	-0.05	0.10	0.13	0.16
Silicon	Si	-0.15	0.61	-0.11	0.40	0.46	-0.08	0.23	0.10	-0.05	0.01
Yttrium	Y	0.54	0.68	0.01	0.01	-0.15	-0.01	0.00	0.08	0.08	0.01
Zirconium	Zr	0.16	0.73	0.18	0.21	-0.03	-0.13	-0.16	0.10	-0.17	0.19
Silicate	431	0.58	-0.59	0.35	0.11	-0.14	-0.15	0.13	-0.09	-0.10	0.21
Quartz	462	0.25	-0.69	0.54	0.12	-0.08	-0.03	0.09	0.05	-0.03	0.18
Copper	Cu	0.45	-0.46	0.07	-0.26	-0.26	0.08	0.25	-0.07	0.10	0.07
Phosphorous	P	-0.26	-0.82	0.17	0.03	-0.25	0.03	-0.14	-0.08	-0.03	-0.24
Silt+clay	SC	0.67	-0.69	0.00	-0.08	0.00	-0.11	-0.06	0.06	-0.02	0.10
Quartz	449	0.21	-0.61	0.68	0.05	-0.15	-0.19	0.09	-0.01	-0.02	0.10

Quartz	777	-0.04	0.31	0.82	-0.19	0.25	-0.21	0.05	-0.12	0.07	-0.12
Quartz	798	-0.21	0.11	0.88	-0.21	0.19	-0.18	0.06	-0.07	0.09	-0.11
Quartz	1081	-0.56	0.00	0.69	-0.26	0.10	-0.30	0.07	0.06	0.06	-0.06
Quartz	1094	-0.65	-0.05	0.68	-0.19	0.07	-0.23	0.05	0.06	0.04	-0.01
Carboantes	2520	0.39	0.20	0.67	-0.06	-0.06	0.18	-0.11	0.07	0.02	-0.23
Aliphatic OM	2842	0.58	0.20	0.66	0.06	-0.02	0.30	0.02	0.03	-0.07	0.01
Aliphatic OM	2853	0.56	0.28	0.59	-0.10	-0.02	0.20	-0.17	0.11	-0.04	-0.03
Aliphatic OM	2879	0.60	0.15	0.64	0.00	-0.01	0.30	-0.06	0.02	0.02	0.04
Aliphatic OM	2922	0.51	0.39	0.57	0.01	-0.02	0.35	-0.04	-0.08	-0.23	0.12
Potassium	K	-0.18	0.07	0.67	0.48	-0.10	-0.03	0.10	-0.03	-0.17	0.13
Aluminum	Al	0.35	-0.10	0.06	0.63	0.11	-0.25	0.37	0.00	-0.09	-0.30
Chromium	Cr	-0.02	-0.29	-0.08	-0.62	0.52	0.24	-0.01	-0.01	-0.27	0.10
Zinc	Zn	0.47	0.43	-0.11	-0.34	-0.52	-0.02	0.18	0.00	0.08	-0.05
Uranium	U	-0.43	-0.23	-0.08	0.38	0.36	0.10	-0.46	0.03	0.00	-0.03
Nickel	Ni	0.11	0.14	-0.03	-0.10	0.08	0.14	0.13	-0.68	0.53	0.16

SI_Table 2. Radiocarbon dates from A Lanzada. Conventional radiocarbon dates were calibrated using the Oxcal v4.4 (C. Bronk Ramsey) and the "INTCAL20" dataset. The extraction of collagen from the bone samples was done with alkali.

Sample	Lab code	$\delta^{13}C$	^{14}C age ± (1σ) yr BP	Calibrated age (2σ), yr AD	Probability	Type of sample
SQ1.S27	Beta-512594	-26.0 ‰	1940 ± 30	10-204	95.4%	Soil
L01	Beta-447670	-18.6 ‰	1410 ± 30	597-664	95.4%	Bone
L06	Beta-452304	-17.8 ‰	1460 ± 30	564-650	95.4%	Bone

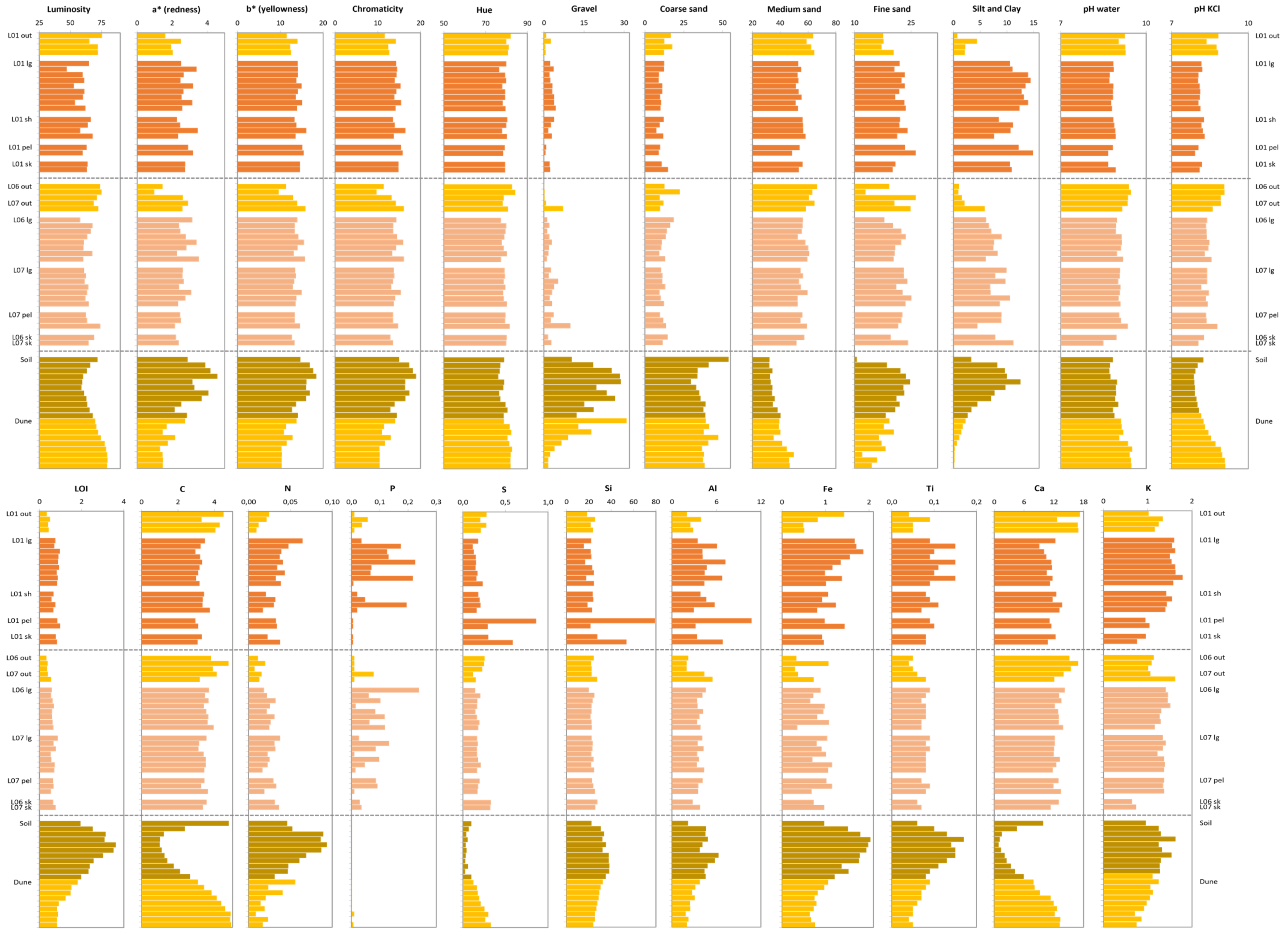
Luminosity
a* (redness)
b* (yellowness)
Chromaticity
Hue
Gravel
Coarse sand
Medium sand
Fine sand
Silt and Clay
pH water
pH KCl
L01 out
L01 lg
L01 sh
L01 pel
L01 sk
L06 out
L07 out
L06 lg
L07 lg
L07 pel
L06 sk
L07 sk
Soil
Dune
LOI
C
N
P
S
Si
Al
Fe
Ti
Ca
K

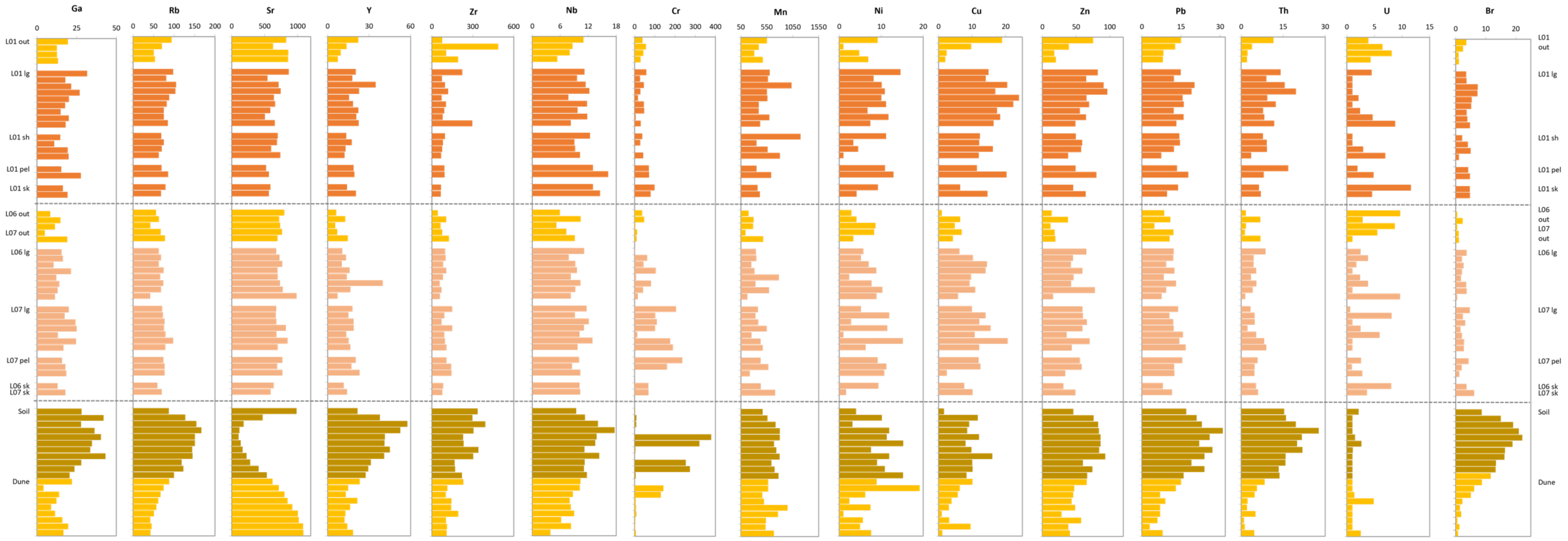

SI_Figure 1. Results for the physicochemical properties and elemental composition. (Grain size distribution, LOI, C, N, P, S, Si, Al, Fe, Ti, Ca and K are expressed in %, the rest of the chemical elements concentration are expressed as μg g-1). The burial samples have been grouped according to the place they were taken (out: outside the burial), the transect (lg: longitudinal) and the anatomical region (sk: skull; sh: shoulders; pel: pelvis) with which they were related.
